# Genetic polymorphism in HTR2A rs6313 is associated with internet addiction disorder

**DOI:** 10.3389/fpsyt.2024.1292877

**Published:** 2024-02-14

**Authors:** Yu Dai, Chenchen Zhang, Lingrui Zhang, Chao Wen, Hongya Li, Tianmin Zhu

**Affiliations:** ^1^ Department of Traditional Chinese Medicine, Chengdu Eighth People’s Hospital (Geriatric Hospital of Chengdu Medical College), Chengdu, China; ^2^ College of Rehabilitation and Health Preservation, Chengdu University of Traditional Chinese Medicine, Chengdu, China; ^3^ Department of Rehabilitation, Traditional Chinese Medicine Hospital of Longquanyi District, Chengdu, China; ^4^ Department of Medicine, Leshan Vocational and Technical College, Leshan, China

**Keywords:** internet addiction disorder, 5-HT receptor, serotonin 2A receptor, rs6313 gene, gene polymorphism

## Abstract

**Introduction:**

Internet addiction disorder (IAD) has grown into public health concern of global proportions. Previous studies have indicated that individuals with IAD may exhibit altered levels of serotonin and dopamine, which are known to play crucial roles in depression, anxiety, impulsivity, and addiction. Therefore, polymorphisms in the receptors that mediate the effects of serotonin and dopamine and affect their functional states as well as their activities are suspect. In this study, we aimed to investigate the association between IAD and rs6313 (T102C) polymorphism in the serotonin 2A receptor (5-HT2A) gene, (HTR2A).

**Methods:**

Twenty patients with IAD and twenty healthy controls (HCs) were included in this study. Young’s Internet Addiction Test (IAT), Self-Rating Anxiety Scale, Self-Rating Depression Scale, Yale-Brown Obsessive-Compulsive Scale (Y-BOCS), Barratt Impulse Scale, Pittsburgh Sleep Quality Index (PSQI), and Social Support Rating Scale (SSRS) were used to assess the severity of internet addiction, mental status, impulsive traits, sleep quality, and social support. Genotyping was performed to identify rs6313 polymorphisms in the HTR2A gene of all participants.

**Results:**

The frequencies of the C and T alleles of HTR2A T102C were 28% and 72% in the IAD group and 53% and 47% in the HCs group, respectively, indicating that the differences between these two groups were significant. No significant difference was observed in the distribution of the CC, CT, and TT genotypes of HTR2A gene T102C between the IAD and the HCs groups. Additionally, there was no difference in the distribution of the frequencies of the HTR2A gene T102C CC and CT+TT genotypes between the two groups. However, the distribution between the TT and CC+CT genotypes showed an apparent statistical difference in the HTR2A gene T102C between the two groups. Correlation analysis indicated that the IAT score was positively correlated with the Y-BOCS and BIS scores for the CC+CT genotype in patients with IAD. Moreover, the IAT score was positively correlated with the PSQI score in patients with IAD carrying the TT genotype.

**Conclusion:**

The present study demonstrates that rs6313 in HTR2A is associated with IAD, and that the T allele of rs6313 in HTR2A may be a risk factor for IAD.

## 1 Introduction

The advent of internet technology has made life, study, and work more convenient for young people. However, inappropriate internet usage may become addictive leading to periodic or chronic indulgence which may harm the physical and mental health of those involved, and weaken their interpersonal communication skills, and social relationships (1, 2). This condition has been termed as Internet addiction disorder (IAD) or Pathological internet use, defined as a behavioral addiction (3). Currently, IAD is considered as a serious public health concern due to its high prevalence worldwide (4–6).

Many scholars who investigated internet addiction from the perspectives of neuroimaging and neurobiology have suggested that IAD may be closely correlated with the dopaminergic and serotoninergic systems. Weinstein et al., reported that compulsive behavior and loss of control in individuals with IA were associated with low levels of dopamine D2 and 5-HT2A receptors in the orbitofrontal cortex (7). Moreover, a study conducted by Dresp–Langley revealed that dysregulation of the serotonin (5-HT) and dopamine (DA) neurotransmitter pathways may be the mechanism underlying IAD (8). Liu et al., reported a significant difference between the levels of dopamine in the peripheral blood of individuals with IAD and healthy participants (9), while Luo et al. (10), found that the levels of 5-HT in the platelets of individuals with IA were different from those of healthy controls. Additionally, Ariatama (11) and Hou (12) found that individuals with IA displayed lower dopamine transporter (DAT) concentrations.

Importantly, 5-HT and DA reportedly play crucial roles in depression, anxiety, impulsivity, and addiction (13–18). Therefore, genes involved in the dopaminergic or serotonergic systems may be regarded as potential candidate genes associated with IAD.

The effects of 5-HT are mediated by the corresponding receptor. Human 5-HT receptors (5-HTR) can be categorized into seven types: 5-HT1 ~ 7R. The serotonin 2A receptor (HTR2A) is a subtype of 5-HTR. Myers study reported that the HTR2A expression is regulated at the gene level (19). This indicates that receptor polymorphisms may affect the functional state of receptors, thereby influencing the activity of 5-HT. Current research on HTR2A gene polymorphism mainly focuses on two SNPs, rs6313 (T102C) and rs6311 (-1438A/G) (20–22). A meta-analysis by Cao et al. (23), suggested that rs6313 in HTR2A was associated with substance use disorders. Several studies have demonstrated that HTR2A is associated with depression, anxiety, sleep, impulsive actions, and cognitive control (24–30). Previous studies have confirmed that depression and anxiety are risk factors for the development of IA (31, 32), and that self-control plays an important role in the generative mechanism of IA (33, 34). Besides, sleep problems is also a common symptom of IA (35). Hence, we hypothesized that the polymorphism in HTR2A rs6313 may be associated with IAD as well as with related comorbidities, such as depression, anxiety, impulsivity, and sleep problems. In this study, we tested the above hypothesis by evaluating the following: 1. the relationship between IAD and rs6313 polymorphism in HTR2A; 2. the association between IAT scores and clinical scores of different genotypes in patients with IAD.

## 2 Materials and methods

### 2.1 Subjects

G*Power was used to evaluate sample size. Chi-square test, with w = 0.5, power = 0.85, and df = 1 was used to analyze results. G*Power indicated that a sample size of 36 would be appropriate. With due consideration given to the dropout rate, 40 participants were recruited from the University of Electronic Science and Technology, Chengdu University of Traditional Chinese Medicine, and the Sichuan Vocational and Technical College of Communication. The participants included 20 patients with IAD and 20 HCs. Beard’s Diagnostic Questionnaire was used to diagnose IAD (36). All participants in our study were right-handed, and none reported any other organic or mental illnesses. In addition, IAD individuals had not undergone any form of therapeutic intervention. This study was subjected to ethical scrutiny and approved by the Ethics Review Board of the Affiliated Hospital of Chengdu University of Traditional Chinese Medicine (Permission number: 2016KL-005). Signed informed consent was obtained from all participants.

### 2.2 Clinical measures

#### 2.2.1 Young’s internet addiction test

The IAT measures the degree of IAD and consists of 20 items (37). The possible scores range from 20 to 100. Moreover, IAT has been confirmed as having adequate validity and reliability. The Cronbach’s alpha was 0,93 (38).

#### 2.2.2 Self-rating depression scale

The SDS consists of 20 self-report questions, which are used to assess depression symptoms (39). Previous studies have shown that the SDS has adequate reliability and validity (40, 41).

#### 2.2.3 Self-rating anxiety scale

The SAS is widely used to ascertain the anxiety state of an individual (42). It consists of 20 items. The Cronbach’s alpha for this scale was 0,883 (43).

#### 2.2.4 Yale-brown obsessive-compulsive scale

The Y-BOCS consists of 10 core items that test the severity of obsessive-compulsive symptoms (44). The Chinese version of the Y-BOCS was found to have adequate reliability in Chinese sample studies, with a Cronbach’s alpha of 0,83 (45).

#### 2.2.5 Barratt impulsiveness scale

The BIS-11 is a self-reported questionnaire consisting of 30 items that evaluates the impulsivity of individuals (46). The Chinese version of the BIS-11 has previously been verified as reliable, with a Cronbach’s alpha of 0,80 (47).

#### 2.2.6 Pittsburgh sleep quality index

The PSQI is widely used to assess the subjective sleep quality of individuals in clinical settings, and consists of 19 items (48). The Cronbach’s alpha for this scale was 0,81 (49).

#### 2.2.7 Social support rating scale

The SSRS was developed by Xiao and consists of ten items (50). This scale is a brief measure of social support and is widely used in Chinese samples (51, 52).

### 2.3 Blood sample collection and preservation

The 5 mL of elbow venous blood was collected and stored in ethylenediaminetetraacetic acid (EDTA) anticoagulant tubes. Upon receipt, the blood samples were stored at −80°C until used.

### 2.4 Genome DNA extraction

Genomic DNA was extracted using the Blood Gene Mini Kit (200 µL, CWBIO, Beijing, China): (1) Approximately 10ˆ^5^ whole blood mononuclear cells were added to 200 µL Buffer GR, treated with 20 µL Protein K and mixed very gently; (2) next, 200 µL GL was added to the preparation, which was then mixed for 15 s, and incubated at 56°C for 10 min, and treated with 200 µL anhydrous ethanol; (3) the solution obtained via the previous steps was added to the adsorption column, and centrifuged at 12000 rpm for 1 min, following which the waste liquid was poured into a collection tube; (4) next, 500 µL of GW1 buffer was added to the adsorption column, which was then centrifuged at 12000 rpm for 1 min, the waste liquid being poured into the collection tube. Subsequently, 500 µL of GW2 buffer was added to the adsorption column, centrifuged at 12000 rpm for 1 min, and the waste liquid poured into the collection tube; (5) next, the solution in the adsorption column was centrifuged at 12000 rpm for 2 min. Immediately afterwards, the adsorption column was placed in a clean centrifuge tube, and let stand at room temperature for a few minutes until the residual liquid in the adsorption column evaporated; (6) approximately, 50-200 µL of GE buffer was added to the adsorption column, let stand at room temperature for 5 min, and centrifuged at 12000 rpm for 2 min. The DNA solution resulting was cryopreserved at −20°C; (7) DNA fragments were detected via 1% agarose gel electrophoresis.

### 2.5 PCR amplification

A reaction volume of 25 μL/tube (2*TaqMan Realtime PCR Mix 12.5 μL + PCR primer pair 1.2 μL + ddH2O 8.3 μL and cDNA template 3 μL) was used. PCR amplification was performed on an FTC-3000QPCR system (Funglyn Biotech, Toronto, Canada). The amplification conditions were as follows: pre-denaturation at 95°C for 10 min; denaturation at 95°C for 10 s; annealing at 55°C for 30 s; extended at 72°C for 30 s; repetition of cycle for 35 times; a final extension at 72°C for 5 min. The following primers were used: forward, CAGCCTCAGTGTTACAGAGT; and reverse, CAGCAATAGTTAGAATAATCACT. The PCR products so obtained were subjected to sequencing (Please refer to our **Supplementary Materials** for the relevant electrophoresis and sequencing diagrams).

### 2.6 Statistical analyses

Statistical analyses were conducted using SPSS 21.0 and GraphPad Prism 7.0 software. The Chi-square test was used to evaluate differences between the genotypes and allele frequencies of the two groups. An independent samples *t*-test or a non-parametric test, was used to detect differences in scale scores between the two groups. For correlation analysis, the data were tested for *K-S* normality. The Pearson correlation coefficient was used to analyze normal distribution, and the Spearman correlation coefficient was used to analyze non-normal distribution. *P ≤ 0.05* was considered to be statistically significant.

## 3 Results

### 3.1 Demographic and clinical measures

**Figure 1:**
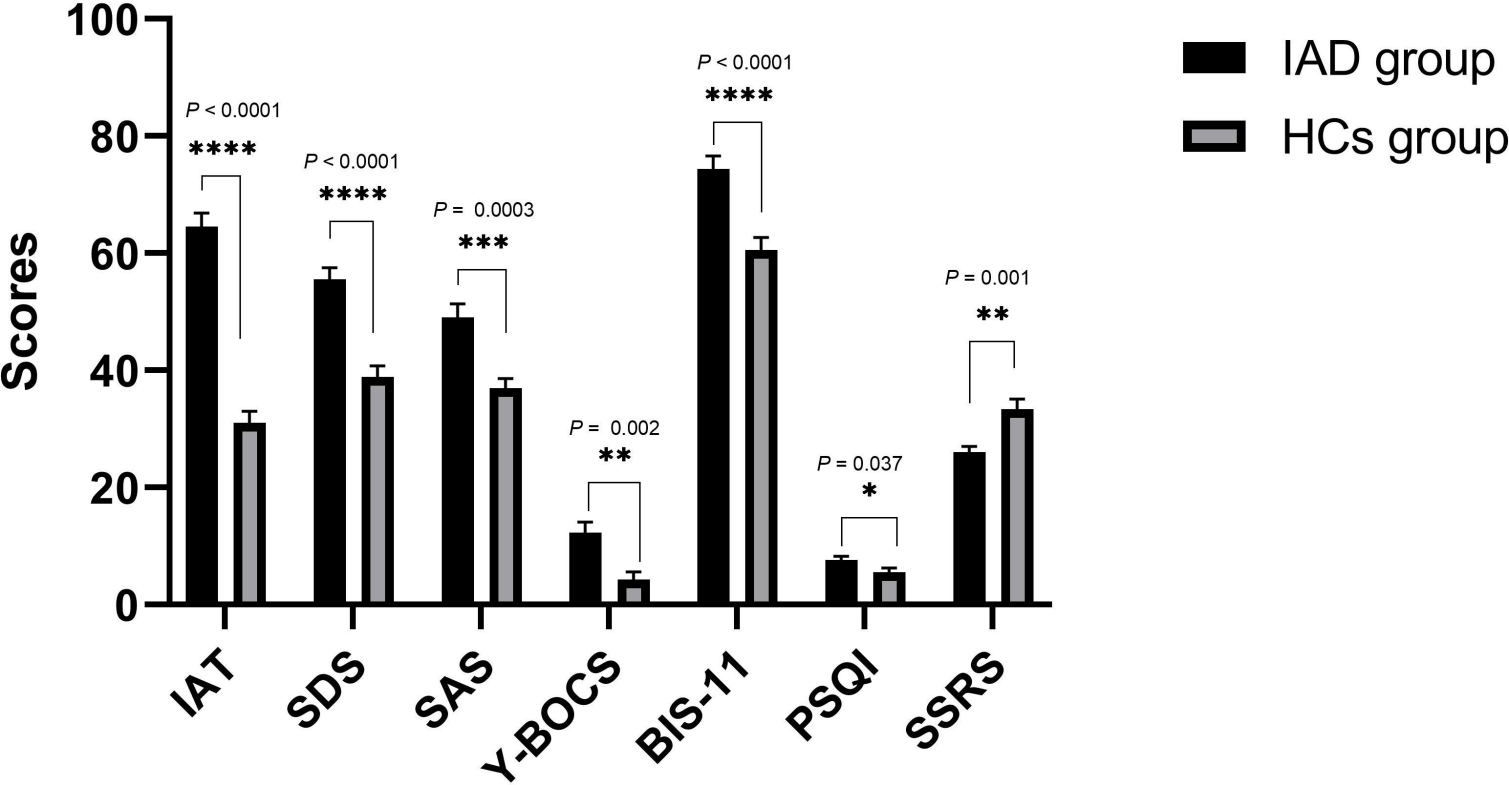
Clinical measures in the IAD group and the HCs group. Bars represent means, error bars represent SEM, triangles and circles represent individual data points. **** *P* < 0.0001, *** *P* < 0.0005, ** *P* < 0.01, * *P* < 0.05. IAT, Young’s Internet Addiction Test; SDS, Self-Rating Depression Scale; SAS, Self-Rating Anxiety Scale; Y-BOCS, Yale-Brown Obsessive-Compulsive Scale; BIS-11, Barratt Impulse Scale; PSQI, Pittsburgh Sleep Quality Index; SSRS, Social Support Rating Scale.

Forty participants consented to take part in the study, of which 20 (100%) patients with IAD and 18 (90%) HCs had valid genetic data. The average age of the 20 patients with IAD (15 male and 5 female) was 20.00 ± 3.00 years, while that of the 18 HCs (14 male and 4 female) was 21.83 ± 2.43 years. There were no significant differences between the ages (*P* = 0.841, *χ2 = *0.04) or sexes (*P* = 0.374, z = -0.888) of the two groups. As shown in **Figure 1**, compared with HCs, patients with IAD had higher IAT, SDS, SAS, Y-BOCS, BIS-11, and PSQI scores. However, the SSRS scores of patients with IAD were lower than those of HCs.

### 3.2 Allele frequency and genotype distribution

The genotype distributions of T102C of the HCs group followed the Hardy-Weinberg equilibrium (*χ2 = *0.919; *P* = 0.337), and the genotype distributions of T102C of the IAD group also followed the Hardy-Weinberg equilibrium (*χ2 = *0.298; *P* = 0.58). **Table 1** shows that the C allele frequencies in the IAD group and the HCs group were 0.28 and 0.53, respectively, and the T allele frequencies in the IAD group and the HCs group were 0.72 and 0.47, respectively. There were differences between the allele distributions in the two groups (*P* ≤ 0.05). As shown in **Table 2**, there was no difference in the distribution of the CC, CT, and TT genotypes of T102C in HTR2A between the IAD and HCs groups (*P* > 0.05). Additionally, there was no difference in the distribution of the frequencies of CC and CT+TT genotypes of T102C in HTR2A in the two groups (*P* > 0.05). However, a statistical difference was observed in the frequency distributions of the TT and CC+CT genotypes of T102C in HTR2A between the IAD group and the HCs group (*P* ≤ 0.05).

**Table 1:** Comparison of allele frequency between the IAD group and the HCs group.

Group	Case	Allele		*χ2*	*P*	OR[95%CI]
		C	T			
IAD	20	11(0.28)	29(0.72)	5.067	0.024	0.34(0.13-0.88)
HCs	18	19(0.53)	17(0.47)			

**Table 2:** Comparison of genotype frequency between the IAD group and the HCs group.

Genotype	IAD (*n* =20)	HCs (*n* =18)	OR[95%CI]	*P*	Adjusted OR[95%CI]^b^	*P^b^*
CC	2(0.10)	4(0.22)	1.00		1.00	
CT	7(0.35)	11(0.61)	1.27 (0.18-8.89)	0.808	1.13 (0.146-8.76)	0.906
TT	11(0.55)	3(0.17)	7.33 (0.88-61.33)	0.066	5.84(0.33-103.34)	0.228
CC	2(0.10)	4(0.22)	1.00		1.00	
CT+TT	18(0.9)	14(0.78)	2.57(0.41-16.12)	0.302	1.14(0.29-14.01)	0.474
CC+CT	9(0.45)	15(0.83)	1.00		1.00	
TT	11(0.55)	3(0.17)	6.11(1.34-27.96)	0.014	5.78(1.18-28.35)	0.03

b Adjusted for age and gender.

### 3.3 Relationship between the IAT score and clinical scores in the CC+CT genotype or the TT genotype in patients with IAD

**Figure 2:**
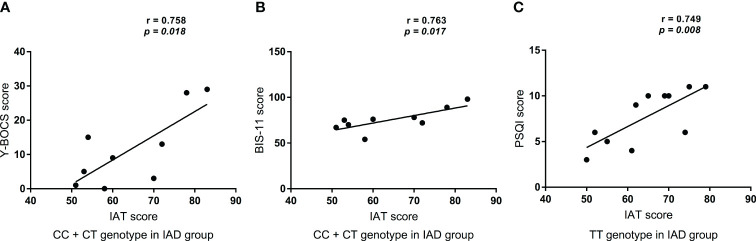
Correlations between IAT score and Clinical scores. **(A)** The relationship between IAT score and Y-BOCS score in CC+CT genotype in patients with IAD (*n* = 9); **(B)** The relationship between IAT score and BIS-11 score in CC+CT genotype in patients with IAD (*n* = 9); **(C)** The relationship between IAT score and PSQI score in TT genotype in patients with IAD (*n* = 11).

There was no correlation between the IAT scores and SDS (*r* = 0.553, *P* = 0.123), SAS (*r* = 0.662, *P* = 0.052), PSQI (*r* = 0.165, *P* = 0.672), or SSRS (*r* = -0.372, *P* = 0.324) scores of the CC+CT genotype in patients with IAD. Moreover, no significant association was observed between the IAT score and the SDS (*r* = 0.566, *P* = 0.069), SAS (*r* = 0.48, *P* = 0.135), Y-BOCS (*r* = 0.54, *P* = 0.087), BIS (*r* = 0.386, *P* = 0.241), or SSRS (*r* = 0.109, *P* = 0.75) scores of the TT genotype in patients with IAD. However, as shown in **Figures 2A** and **B**, the IAT score was positively correlated with the Y-BOCS (*r* = 0.758, *P* = 0.018) and BIS scores (*r* = 0.763, *P* = 0.017) of the CC+CT genotype in patients with IAD. Additionally, **Figure 2C** shows the IAT score was positively correlated with the PSQI score (*r* = 0.749, *P* = 0.008) of the TT genotype in patients with IAD.

## 4 Discussion

IAD, also referred to as pathological internet use or overuse, has come to be considered as a serious global public health issue. Internet addiction often manifests as excessive use of the internet, or an inability to fully concentrate on daily activities, or to effectively control desires and behaviors related to internet use, thereby adversely affecting all aspects of life (53). Depression and anxiety are the most common psychological disorders that have been found to be associated with IAD (32). Individuals beset with negative emotions resort to the internet to cope with psychological issues. If internet use relieves their negative emotions even temporarily, they may become more prone to excessive internet use, with such interdependency quickly turning into a vicious cycle of negativity (54). The present study indicated that patients with IAD had higher SDS and SAS scores than HCs. A previous review revealed that the reward system plays a crucial role in IAD (7). Similarly, in our previous study, we found that IAD was associated with reward systems (55). Moreover, the 5-HT system participates in the regulation of brain reward function (56). Abnormal expression of the 5-HT system is often associated with impulsive behavior (57) and sleep problems (58), which are common symptoms of IAD. A recent animal experiment reported that 5-HT levels may affect social interactions during protracted opioid withdrawal in mice (59). Our study showed that patients with IAD had higher Y-BOCS, BIS-11, and PSQI scores than HCs. In addition, patients with IAD had lower SSRS scores than healthy participants.

Overall, the 5-HT system plays a crucial role in IA. Several genetic studies have explored IAD. According to one review, the main candidate genes involved in IAD included those encoding the dopamine D2 receptor, monoamine-oxidase-A, and serotonin transporter (5-HTT) (60). HTR2A is a subtype of 5-HTR, which affect 5-HTT function and serotonergic transmission (61, 62), and has thus turned into a research hotspot in substance addiction, aggressive behavior, and obsessive-compulsive disorder in recent years (63–65). Mounting evidence has implicated serotonin neurotransmission through the 5-HT2A receptor as a driver of relapse-related behavior in substance addiction (66, 67). HTR2A, which consists of two introns and three exons, is located on chromosome 13q14-q21. It encodes a G protein-coupled receptor associated with phospholipase, which is mainly distributed in the frontal cortex, hippocampus, amygdala, and peripheral blood. Recent research aimed at the association between HTR2A polymorphisms and addiction has mainly focused on rs6313 (polymorphism rs6313 is located in codon 102 of 5-HTR2A exon 1) (23, 68, 69).

A previous study on Mexican mestizos showed that the T allele carrier of the single nucleotide polymorphism rs6313 (102T>C) in HTR2A was associated with the risk of cigarette smoking (21). White et al., reported that the T102C TT genotype (Homozygotes for T102) was a significant predictive risk factor for cigarette smoking (68). In the present study, we conducted a genotyping analysis of T102C in patients with IAD and healthy participants, and found that the TT genotype of rs6313 in HTR2A was associated with an increased risk of IA.

Furthermore, to understand the relationship between different genotypes of rs6313 in HTR2A in patients with IAD and clinical scores, we conducted a correlation analysis and found that the IAT score was correlated with the BIS-11 and Y-BOCS scores in patients with IAD carrying the C allele of rs6313. The IAT score was only correlated with the PSQI score for the TT genotype of rs6313 in patients with IAD. Moreover, 5-HT activity is related to cognition and impulsive behavior (70), while HTR2A is known to regulate 5-HT neurotransmission (71). Polesskaya et al., reported that compared with the T allele, the presence of the C allele in rs6313 reduced the expression of HTR2A by 20% (72). This indicated that changes in the mRNA expression of HTR2A may modulate the function of 5-HT, in turn influencing its cognitive and impulsive effects. Previous studies have shown that the 5-HT2A receptors play a role in sleep (27). Elmenhorst et al., indicated that sleep deprivation increases HTR2A binding in the neocortex of healthy individuals (73). Notably, a recent study reported that preventing mice from sleeping resulted in a significant increase in HTR2A mRNA levels in their cortices (74). One Chinese study reported that although −1438G/A of HTR2A was associated with sleep quality in non-manual workers, T102C was not (26). Japanese researchers have reported that the C allele of HTR2A T102C was associated with sleep bruxism (75). Interestingly, the present study showed a positive correlation between the degree of internet addiction and sleep problems in the TT genotype of rs6313 in patients with IAD. These contradictory results may be attributed to factors such as differences in age and disease status.

This study had several limitations. Our results indicated that the T allele of rs6313 was associated with IAD. However, small sample size limited the generalizability of our current results. In addition, we did not consider the involvement of the SNPs in other genes linked to internet addiction. Therefore, future studies involving larger sample sizes and other genes that are likely to be linked to IAD are warranted.

To the best of our knowledge, this exploratory study is the first to focus on the association between genetic polymorphisms in HTR2A and IAD. Our results suggest that the T allele of rs6313 in HTR2A is a risk factor for IAD. Although our results are of a preliminary nature, they may provide new insights into the pathogenesis of IAD.

## Data availability statement

The original contributions presented in the study are included in the article/**Supplementary Material**. Further inquiries can be directed to the corresponding author.

## Ethics statement

The study underwent ethical scrutiny and was approved by the Ethics Review Board of the Affiliated Hospital of Chengdu University of Traditional Chinese Medicine (Permission number: 2016KL-005). The studies were conducted in accordance with the local legislation and institutional requirements. The participants provided their written informed consent to participate in this study.

## Author contributions

YD: Writing – original draft, Formal Analysis. CZ: Data curation, Writing – review & editing. LZ: Writing – review & editing. CW: Writing – review & editing. HL: Formal Analysis, Writing – review & editing. TZ: Conceptualization, Funding acquisition, Methodology, Writing – review & editing, Supervision.
