# Supplementary figures and images for: Genetic polymorphism in HTR2A rs6313 is associated with internet addiction disorder
Source: Front Psychiatry. 2024 Feb 14;15:1292877. doi: 10.3389/fpsyt.2024.1292877 (PMC10899489; doi:10.3389/fpsyt.2024.1292877)

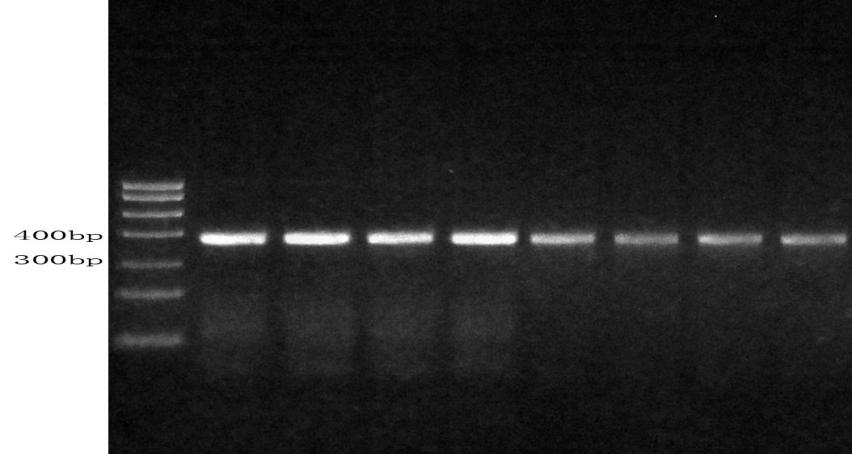


Supplemental 1 Electropherogram (HTR2A)


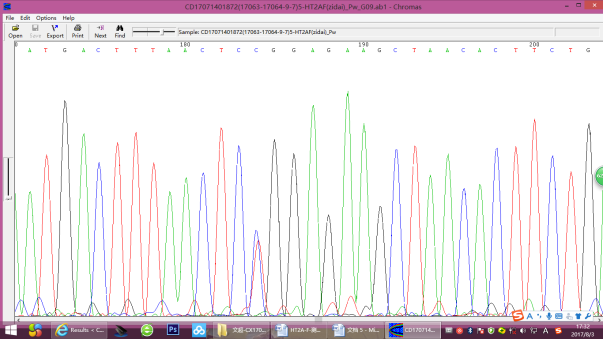

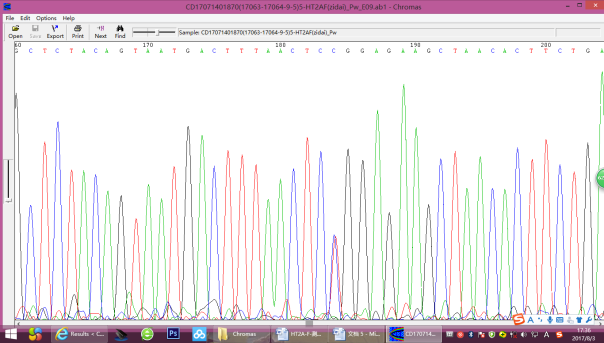


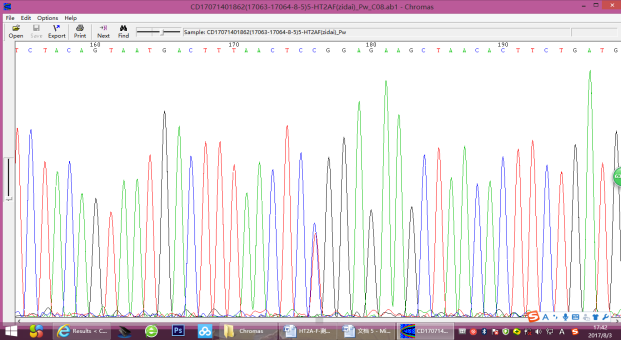

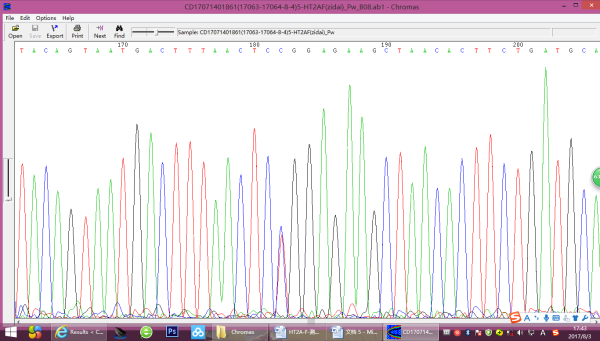


Supplemental 2 Sequencing results - heterozygote

Supplement: Supplementary file 1 [file DataSheet_1.docx]
